# Implementation of a genomic medicine multi-disciplinary team approach for rare disease in the clinical setting: a prospective exome sequencing case series

**DOI:** 10.1186/s13073-019-0651-9

**Published:** 2019-07-25

**Authors:** John Taylor, Jude Craft, Edward Blair, Sarah Wordsworth, David Beeson, Saleel Chandratre, Judith Cossins, Tracy Lester, Andrea H. Németh, Elizabeth Ormondroyd, Smita Y. Patel, Alistair T. Pagnamenta, Jenny C. Taylor, Kate L. Thomson, Hugh Watkins, Andrew O. M. Wilkie, Julian C. Knight

**Affiliations:** 1 Oxford Centre for Genomic Medicine, Oxford University Hospitals NHS Foundation Trust, Oxford, UK; 2 Nuffield Department of Population Health, University of Oxford, Oxford, UK; 3 MRC Weatherall Institute of Molecular Medicine, University of Oxford, Oxford, UK; 4 Children’s Hospital, Oxford University Hospitals NHS Foundation Trust, Oxford, UK; 5 Nuffield Department of Clinical Neurosciences, Oxford University Hospitals NHS Foundation Trust, Oxford, UK; 6 Division of Cardiovascular Medicine, Radcliffe Department of Medicine, University of Oxford, Oxford, UK; 7 National Institute for Health Research Biomedical Research Centre, Oxford, UK; 8 Department of Clinical Immunology, Oxford University Hospitals NHS Foundation Trust, Oxford, UK; 9 Wellcome Centre for Human Genetics, University of Oxford, Oxford, UK

**Keywords:** Genetic disease, Genome sequencing, Exome, Multidisciplinary team, Next-generation sequencing

## Abstract

**Background:**

A multi-disciplinary approach to promote engagement, inform decision-making and support clinicians and patients is increasingly advocated to realise the potential of genome-scale sequencing in the clinic for patient benefit. Here we describe the results of establishing a genomic medicine multi-disciplinary team (GM-MDT) for case selection, processing, interpretation and return of results.

**Methods:**

We report a consecutive case series of 132 patients (involving 10 medical specialties with 43.2% cases having a neurological disorder) undergoing exome sequencing over a 10-month period following the establishment of the GM-MDT in a UK NHS tertiary referral hospital. The costs of running the MDT are also reported.

**Results:**

In total 76 cases underwent exome sequencing following triage by the GM-MDT with a clinically reportable molecular diagnosis in 24 (31.6%). GM-MDT composition, operation and rationale for whether to proceed to sequencing are described, together with the health economics (cost per case for the GM-MDT was £399.61), the utility and informativeness of exome sequencing for molecular diagnosis in a range of traits, the impact of choice of sequencing strategy on molecular diagnostic rates and challenge of defining pathogenic variants. In 5 cases (6.6%), an alternative clinical diagnosis was indicated by sequencing results. Examples were also found where findings from initial genetic testing were reconsidered in the light of exome sequencing including *TP63* and *PRKAG2* (detection of a partial exon deletion and a mosaic missense pathogenic variant respectively); together with tissue-specific mosaicism involving a cytogenetic abnormality following a normal prenatal array comparative genomic hybridization.

**Conclusions:**

This consecutive case series describes the results and experience of a multidisciplinary team format that was found to promote engagement across specialties and facilitate return of results to the responsible clinicians.

**Electronic supplementary material:**

The online version of this article (10.1186/s13073-019-0651-9) contains supplementary material, which is available to authorized users.

## Background

There are unprecedented opportunities for advancing clinical practice through the application of next-generation sequencing (NGS), reflected in rapid adoption by specialist clinics for diagnostic purposes in suspected rare genetic disorders [1–6]. However, there is recognition that while rapid technological advances and reducing cost have made adoption of genome-scale NGS a realistic goal, effective implementation into the clinic for direct patient benefit remains challenging, with many current barriers to widespread adoption. These range from a demonstration of improvement in patient outcomes and cost-effectiveness, to practical difficulties involving physician support and education, establishing pathogenicity for identified variants, handling large complex datasets, dealing with secondary findings whether incidental or sought, and managing the expectations of the patient, clinician and general population [7].

To address these barriers, a number of approaches are advocated including engagement and support of clinicians responsible for individual patients’ care, appropriate case selection and relevant phenotyping, adoption of the most appropriate sequencing strategy for the individual and family, establishing mechanisms for informed consent, implementation of effective sample and bioinformatic pipelines, and support for interpretation of results by both the clinician and patient. Moreover, to realise its potential, the application of genomics in rare disease requires adoption outside of traditional specialties such as clinical genetics and a cross-disciplinary approach among clinical practitioners and allied professionals.

These challenges to implementation will vary in different healthcare settings, and sharing experience and strategic approaches is important. The large consecutive case series reported to date have been predominantly from the USA, including the experience of Baylor College of Medicine [1, 2], the University of California Los Angeles [8] and the Undiagnosed Diseases Network [9], with most cases involving nervous system dysfunction, notably developmental delay, and overall molecular diagnosis rates of 25–35%. Here we describe our experience of establishing a genomic medicine multi-disciplinary team (GM-MDT) in Oxford, UK, through the National Institutes of Health Research (NIHR) Oxford Biomedical Research Centre (BRC) and subsequent roll-out within the Oxford University Hospitals (OUH) NHS Trust. We aimed to engage and support local clinicians to use NGS for patient benefit within a tertiary referral hospital, providing a mechanism for referral, generation and return of results that built on local expertise in a research setting for variant calling, filtering and annotation with the discovery of clinically actionable variants [3]. We recently described qualitative aspects of decision-making [10] and perspectives of clinical genomics professionals in the context of the GM-MDT toward secondary findings [11]. In this paper, we focus on the operation and impact of the GM-MDT, including a prospective case series involving exome sequencing (ES) and health system costs for running the GM-MDT.

## Methods

### Patient participation

Details of the consent process and qualitative analysis of decision-making in the GM-MDT have been previously described including how, dependent on consent, patients had the option to receive “secondary findings” [10]. Patients participated under the Molecular Genetic Analysis and Clinical studies of Individuals and Families at Risk of Genetic Disease (MGAC) protocol approved by West Midlands Research Ethics Committee, reference number 13/WM/0466.

### Clinical samples

Following written informed consent for genetic testing from the patient and/or their parent/legal representative, or other family member, venous blood was obtained. Genomic DNA was extracted from peripheral blood or tissue. Clinical samples were processed, and sequencing results were validated in the Oxford Molecular Genetics Laboratory. ES was performed at the Wellcome Centre for Human Genetics (WHG), Oxford.

### Exome sequencing and bioinformatic analysis

DNA libraries were prepared from 3 μg patient DNA extracted from whole blood. Exome capture was performed using SeqCap EZ Human Exome Library v2.0 or v3.0 (NimbleGen), according to the manufacturer’s instructions, and sequenced using a 100 bp paired-end read protocol on the HiSeq2500 (Illumina). Exome sequence reads were mapped to the hs37d5 reference genome with Stampy [12]. Variants were called with Platypus version 0.5.2 [13]. The variants were annotated and analysed using VariantStudio version 2.2 (Illumina) for targeted gene-panel analysis or Ingenuity Variant Analysis (Qiagen) for an a priori approach to variant detection. Aligned sequence reads were visualised using Integrative Genomics Viewer (IGV) [14]. Copy number variations were called using ExomeDepth [15]. A molecular diagnosis was considered based on the variant(s) identified, gene(s) involved and the case history. Gene sets for the presenting conditions were established based on pre-existing diagnostic gene panels published on the UK Genetic Testing Network (https://ukgtn.nhs.uk/), which were supplemented with additional genes/targets based on literature searches and established protein-protein interaction networks. Variants with minor allele frequency (MAF) > 1% in dbSNP or Exome Aggregation Consortium (ExAC) were removed, and remaining variants were interpreted by the responsible expert analyst and the GM-MDT by review of the literature, available databases, presenting phenotype, proposed mode of inheritance and American College of Medical Genetics and Genomics (ACMG) guidelines regarding potential pathogenicity [16]. All variants were independently validated by Sanger sequencing using BigDye Terminator kit 3.1 (Applied Biosystems) combined with purification using the Agencourt CleanSEQ system. Capillary electrophoresis was performed using an ABI Prism 3730 Genetic Analyser (Applied Biosystems).

### Costing the MDT process, sequencing and analysis

As most healthcare systems face financial constraints, it is important to consider the resources and associated costs for implementing new programmes, including MDTs. Therefore, staff estimated the amount of time (in hours) spent during GM-MDT meetings, time preparing for the MDTs and any post-meeting follow-up. The GM-MDT meeting times were the same for all staff attending (2 h per meeting). The average cost was then estimated for the 132 cases going through the MDT during the 10-month period. Information on clinical and scientific staff salaries was taken from national salary scales from the Unit Costs of Health and Social Care 2018 and from University of Oxford scales for university staff (see Additional file 1: Tables for details). The mid-points of salary ranges were used, a working year was assumed to be 44 weeks and a working week was assumed to be 37.5 h. National Insurance and Superannuation were added to the salary costs and institutional overheads then added at 20%. Exome sequencing costs were derived from the WHG, and analysis time was recorded by clinical scientist undertaking the analysis. ES and analysis costs were for the 174 samples sequenced (76 probands and 98 family members). Costs are reported in 2019 prices where possible.

## Results

### GM-MDT process and prospective ES cohort

**Fig. 1:**
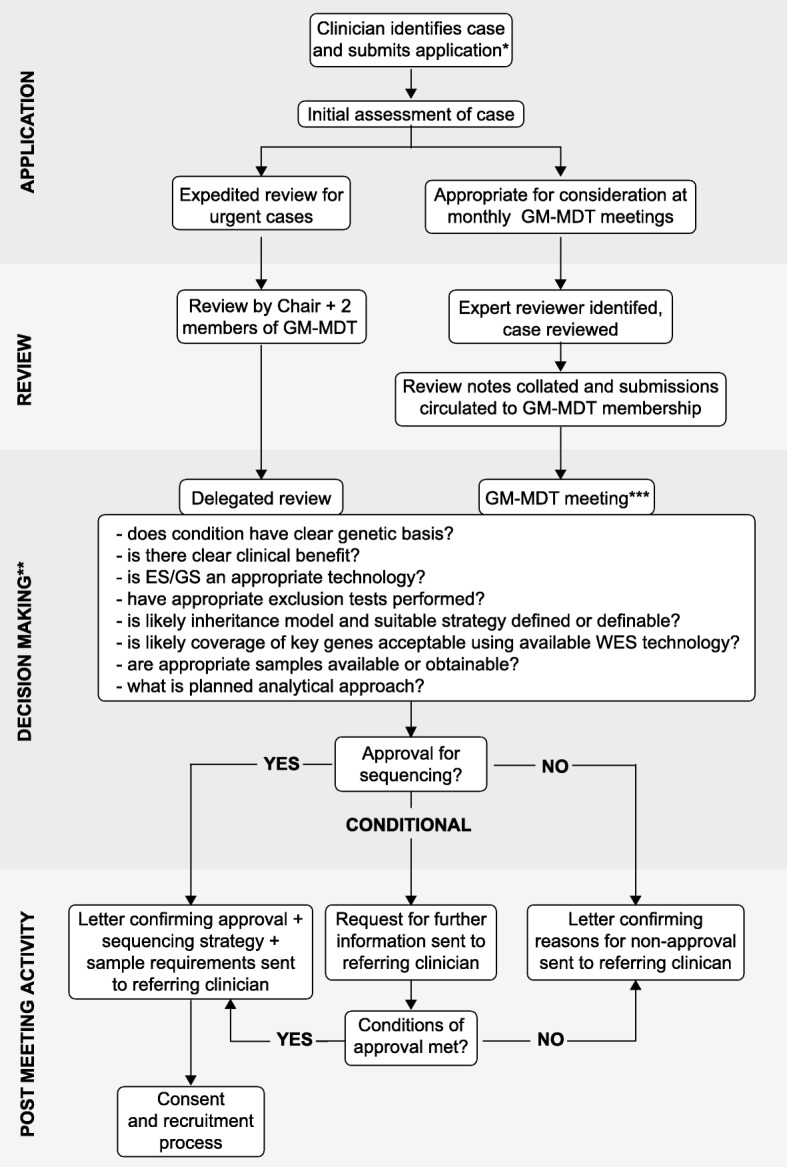
Case review and approval process for GM-MDT. *Application includes clinical phenotype and disease information, demographics, family history including pedigree, ethnicity, evidence or likelihood of consanguinity, prior genetic testing, likely clinical utility/impact on management, genes/variants known to cause the disorder, samples availability and those proposed for genetic testing. **Key questions addressed as part of review process are illustrated; other points often case specific. ***Discussion recorded by project manager in meeting minutes. Figure is based on practice up to the end of October 2015 (including all cases reported here); current process described in Ormondroyd et al. 2017 [10]

The GM-MDT was established as an initiative supported by the Oxford National Institute for Health Research (NIHR) Biomedical Research Centre (BRC) through a process of outreach and education across clinical specialty areas with clinicians participating from 11 specialties (cardiology, clinical genetics, endocrinology, gastroenterology, haematology, immunology, infectious diseases, musculoskeletal diseases, neurology, oncology and renal medicine) together with genetic counsellors, ethicists, bioinformaticians, non-clinical researchers and clinical scientists from the Oxford Molecular Genetics Laboratory (Additional file 1: Table S1). In terms of case submission, requests for sequencing were initiated by, and at the discretion of, the referring clinician who retained clinical responsibility for the patient and actionable results. Expert peer review for each case was provided by a nominated reviewer with a subsequent discussion at the GM-MDT meeting, held monthly on the hospital site (average attendance 14 members) with a fast-track decision-making process for more urgent cases (Fig. 1). The review process and key questions addressed in that decision-making are illustrated (Fig. 1).

**Fig. 2:**
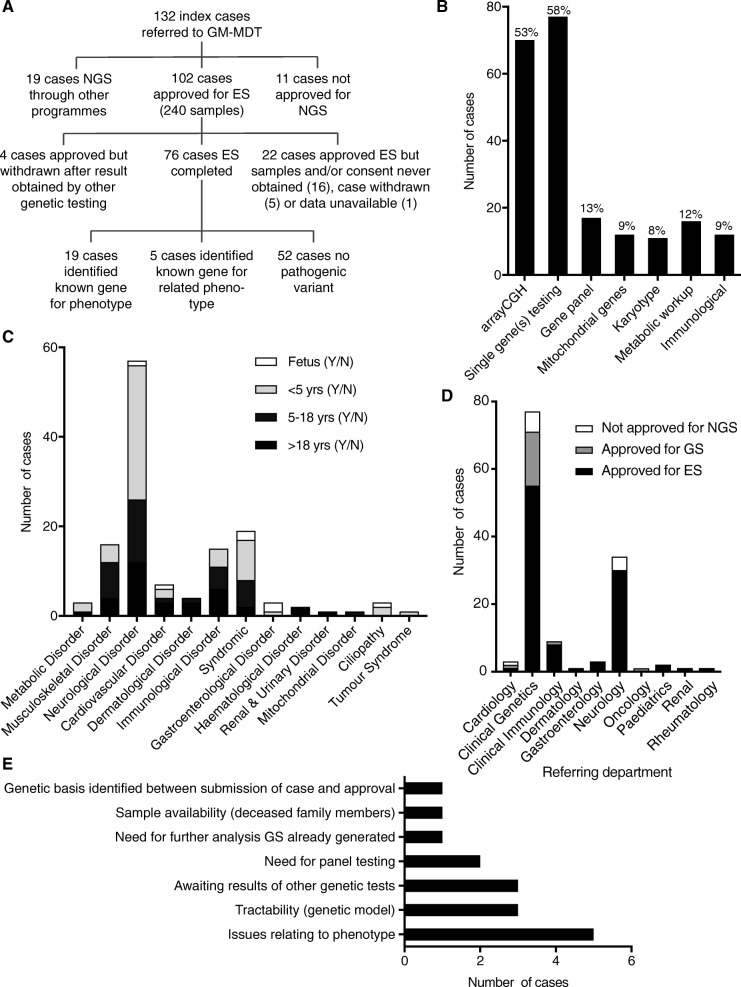
Overview of consecutive cases reviewed by GM-MDT during period April 2014–February 2015. **a** Flow chart describing case allocation for NGS and outcome of ES. **b** Investigation prior to GM-MDT referral. **c** Summary of referrals by class of disorder based on working diagnosis (presenting complaint) and age. **d** Summary of cases by referring department and approval. **e** Reasons for failure to approve cases (more than one may apply to a given case). Issues relating to the phenotype included complexity, variability, issues with affected status of family members and need for formal clinical genetics review

During the initial 10-month period following the establishment of the GM-MDT (May 2014 to February 2015), a total of 132 consecutive cases were submitted (Fig. 2a) (Additional file 1: Table S2). The cases involved rare diseases with a likely monogenic aetiology where there was evidence of potential clinical utility from establishing a genetic basis. Appropriate exclusion of known or likely genetic causes was performed by the time of approval for ES. In some instances, additional genetic testing was recommended by the GM-MDT as a pre-requisite to ES. This most commonly involved array comparative genomic hybridization (aCGH) testing (predominantly cases involving learning difficulties and neurological phenotypes) and single gene(s) sequencing (in 53% and 58.3% of cases respectively) while gene panel testing, mitochondrial gene sequencing, karyotyping, metabolic workup and immunological testing were performed in a minority of cases (Fig. 2b). Similarly, requests for further clinical information on phenotype of the proband or family members (in some instances requiring clinical evaluation, for example to establish affected status) in a minority of cases resulted in deferring decision-making, or less commonly, failure to approve cases (Fig. 1). The cohort comprised 55 children < 5 years of age (41.7% of all cases), 36 children and adolescents 5–18 years of age (27.3%) and 34 adults (25.8%), together with 7 (5.3%) fetal samples. Considering all cases of live births, the geometric mean age was 6 years (95% confidence intervals 4.6–7.8 years) (range 0.1–58 years) and 47.7% of cases were female. The most frequent primary working diagnosis on referral was of neurological disorder (57 cases, 43.2%) with a range of other disorders referred (Fig. 2c). A detailed breakdown by human phenotype ontology is provided in Additional file 2: Figure S1. Referrals were received from 10 clinical departments within the Oxford University Hospitals NHS Trust (Fig. 2d).

A total of 121 cases (91.7%) were approved for NGS of which 102 cases (84.3%) were approved for clinical ES (Fig. 2a). The remaining 19 cases (15.7%) went forward for NGS through other programmes, either locally for genome sequencing (GS) (13 cases) or through national initiatives (6 cases); the latter included the 100,000 Genomes Project (http://www.genomicsengland.co.uk) pilot and the Deciphering Developmental Disorders (DDD) Project [17]. Eleven cases (8.3%) were assessed as not appropriate for NGS following review due to issues in one or more areas (Fig. 2e). Here, the results of the consecutive series of cases taken forward for ES are described together with the estimation of the resources and costs associated with the GM-MDT.

### Results of a consecutive case series subjected to ES

Of the 102 cases (240 samples) approved for ES, this was completed on 76 (74.5%) cases (Fig. 2a). In 16 (15.6%) cases, consent and or samples were never obtained, 5 (4.9%) cases were withdrawn, 4 (3.9%) cases results were obtained by other genetic testing prior to sequencing being performed (Fig. 2a). The geometric mean age of cases where ES results were available was 6.2 years (95% CI 4.1–9.3 years) (range 0.08–56 years) and 46.9% of cases were female. The average BAM file was 8 Gb in size and provided an average gene coverage of 89% at 10× read depth and 80% coverage at 30× read depth across the targeted exome.

We estimated the costs per case for the time spent by staff discussing cases in the MDT, preparation for the meeting reviewing specific cases and meeting follow-up to be £399.61 per case. Similar proportions of time were devoted to the pre-meeting and MDT meetings themselves (37% each), with the remaining time devoted to post-meeting activities (26%) (Additional file 1: Table S3). ES costs were £797 per exome including library preparation and sequence data alignment and variant calling, with associated analysis being £166.60 per case (Additional file 1: Table S4). In our sensitivity analysis, the greatest reduction in costs would come from either a smaller number of individuals on the MDT or having lower grade staff, especially substituting consultants by registrars (as shown in Additional file 1: Table S4). However, the range and depth of experience of the MDT members has clear value in supporting informed decision-making; arguably, some of this benefit would be lost with less experienced individuals.

Overall, the molecular diagnosis rate was 31.6% (24 out of 76 cases), comprising of cases where ES results were judged to be clinically reportable for follow-up by the referring clinician. A detailed summary of findings is provided in Additional file 1: Table S2. We found that 19 (79% of cases where reports issued) involved a known gene for the phenotype while in 5 cases (21%) a known gene for a related phenotype was identified enabling an alternative diagnosis to be considered. Cases of Mendelian disease with a molecular diagnosis included the following proposed modes of inheritance: 12 cases autosomal dominant (of these, all 10 cases for whom parental data were available were de novo, including one mosaic in the affected child), 10 cases autosomal recessive inheritance (6 cases compound heterozygotes, 4 cases homozygous), 1 case X-linked dominant and 1 case X-linked recessive (Table 1).

**Table 1:** Molecular diagnoses in Mendelian diseases among 24 positive cases

Inheritance	Gene	Number of cases
Autosomal dominant^a^	*CACNA1A*, *CHD2*, *FLNC*, *KCNT1*, *KIF11*, *PRKAG2*, *SF3B4*, *SPAST*, *SYNGAP1*, *TCF4*, *TNNT3*, *TP63*	12
Autosomal recessive	*AGRN*, *BRAT1*, *COLQ* (in 2 patients), *CTPS1*, *KPTN*, *LZTR1*, *PAPSS2*, *PTPRC*, *SPG7*	10
X-linked dominant	*WDR45*	1
X-linked recessive	*AIFM1*	1

Inheritance and identified genes are shown

^a^100% de novo (where trio sequenced)

**Fig. 3:**
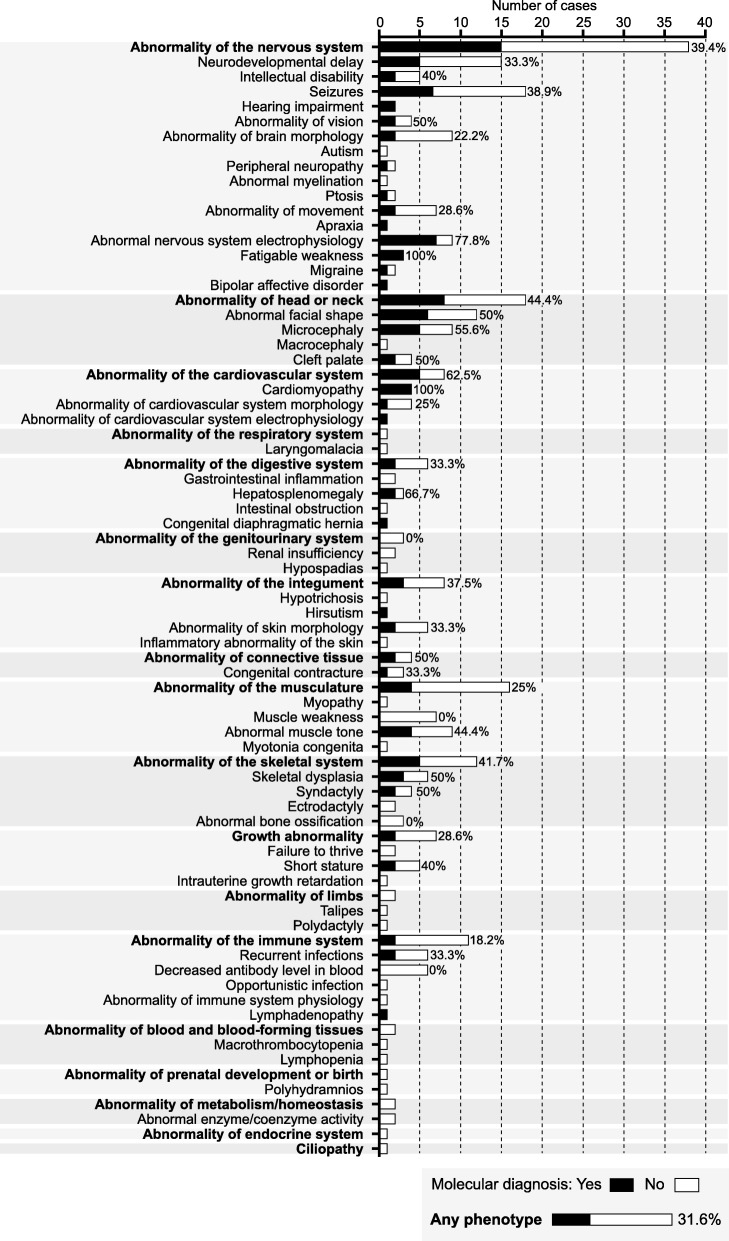
Diagnostic rates by phenotype for ES (*n* = 76 cases). Human phenotype ontology terms generated from clinical presentation. Diagnostic rates are shown (%) together with number of cases for a given phenotype where a molecular diagnosis was made (filled boxes) or no diagnosis made (white boxes)

The highest overall diagnostic rate was seen in children < 5 years of age with a rate of 43.3% (13 out of 30 cases sequenced) (Table 2). The diagnostic rate was higher in patients presenting with neurological traits (40.0%) than in non-neurological traits (24.4%). To explore the relationship between molecular diagnostic rate and phenotype further, we considered human phenotype ontology terms (Fig. 3). This highlighted a number of trends with a higher rate observed in patients with features including seizures (38.9%), neurodevelopmental delay (33.3%) and abnormal nervous system electrophysiology (77.8%) while abnormality of brain morphology and abnormality of movement were associated with lower diagnostic rates (22.2% and 28.6% respectively). Among other phenotypes, higher positive diagnostic rates were seen in cases with abnormal facial shape (50%), microcephaly (55.6%) and cleft palate (50%). In all trios with apparent de novo cases of cardiomyopathy where selection of cases was amenable to a trio design, a molecular diagnosis was made. Cases with abnormality of the skeletal system also had a higher rate (41.7%) including a 50% diagnostic rate in skeletal dysplasia and syndactyly. Cases with abnormal muscle tone as part of the phenotype also had a relatively high diagnostic rate (44.4%).

**Table 2:** Completed cases ES showing rate of molecular diagnosis in terms of age and sequencing strategy

	Molecular diagnosis (*N*)	Total (*N*)	Rate (%)	95% CI
Age				
Fetus	0	4	0.0	–
< 5 years	13	30	43.3	27.4–60.8
5–18 years	6	18	33.3	16.3–56.3
> 18 years	5	24	20.8	9.2–40.5
Sequencing strategy				
Trio^a^	15	43	34.9	22.4–49.8
Singleton	4	18	22.2	9.0–45.2
Other^b^	5	15	33.3	15.2–58.3
All cases	24	76	31.6	22.2–42.7

^a^Proband and both unaffected parents; ^b^in 9 cases proband and affected siblings (7 cases), cousin (1 case) or grandfather (1 case); in the remaining 6 cases the proband alone was sequenced but with unrelated cases having the same phenotype included in this case series (3 singleton cases myaesthenic syndrome, molecular diagnosis in 1 case; 2 cases migralepsy, molecular diagnosis in 1 case)

In total, 174 samples were sequenced, comprising the 76 probands and 98 family members. For 43 cases (56.6% of total), a trio design (affected proband and unaffected parents) was analysed with a molecular diagnosis rate of 34.9% while in 18 cases where a singleton was analysed, the molecular diagnosis rate was 22.2% (Table 2). In some instances, a singleton was screened using a zygosity-filtered approach in the first instance as parents were consanguineous and it was felt that sequencing the parents offered little additional power to the analysis given the likelihood of the variant falling within a region of homozygosity. For example, in a 13-year-old male proband with symptoms consistent with a diagnosis of spondyloepiphyseal dysplasia, a homozygous variant was identified in the *PAPSS2* gene (c.1000C>T, p.(Arg334*)). Subsequently, testing confirmed the variant was inherited from both parents. Homozygous loss of function variants were previously reported to cause spondyloepiphyseal dysplasia [18, 19]; therefore, the variant was considered to be pathogenic.

A further example where ES was performed on a proband alone involved a 54-year-old male with ptosis, cognitive decline, ataxia, cerebellar atrophy, mild hearing impairment, progressive external ophthalmoplegia and bipolar disorder. Previous mitochondrial analysis had not provided a molecular diagnosis. ES revealed the patient had two heterozygote pathogenic variants in *SPG7* (c.1454_1462del, p.(Arg485_487del) and c.1672A>T, p.(Lys558*)). Subsequent analysis of the patient’s parents confirmed the variants were inherited on separate alleles. *SPG7* encodes an ATP-dependent proteolytic complex of the mitochondrial inner membrane reported as pathogenic for chronic progressive external ophthalmoplegia through disordered mitochondrial DNA maintenance [20].

This and other cases also highlighted the utility of ES in enabling simultaneous analysis of genes reported in the literature to be pathogenic for a given condition at the time of ES data being generated. For example, we identified a homozygous variant in the CTP synthase gene *CTPS1* (c.1692-1G>C) in a 4-year-old male with combined immunodeficiency and EBV susceptibility. The same homozygous loss of function variant in this gene, causing similar immunodeficiencies, was described at the time of reporting [21]. Sanger sequencing confirmed heterozygous parents, and a similarly affected sibling was homozygous for the familial variant. This had a significant impact on clinical decision-making, the proband successfully undergoing matched unrelated donor haematopoietic stem cell transplantation [22].

ES can enable successful interrogation of complex cases with a broad differential diagnosis. This is illustrated by a 5-year-old male proband with severe global developmental delay and truncal hypotonia since birth, stroke-like episodes affecting the left and right cerebral hemisphere, seizures and oculomotor apraxia. The patient had been previously screened for a mitochondrial cause. Parent-child trio analysis by ES revealed a de novo variant within the *CACNA1A* gene (c.4043G>A, p.(Arg1348Gln)). This previously reported pathogenic variant [23] could explain the phenotype and illustrates the clinical and phenotypic heterogeneity associated with these conditions and the utility of exome analysis, allowing multiple differential diagnoses to be screened simultaneously. *CACNA1A* variants have been reported in patients with episodic ataxia [24] and spinocerebellar ataxia [25]; however, in this patient, the initial targeted diagnostic screens prior to ES had focused on respiratory chain defects and common mitochondrial mutation analysis (including m.3243A>G, m.8993 T>C, *POLG*). Identification of specific genetic aetiologies within a condition can also have important implications for treatment, as illustrated by this patient for whom treatment with acetazolamide led to significant reduction in episodic symptoms and by our findings in three patients with congenital myasthenic syndrome involving *COLQ* and *AGRN* (Additional file 1: Table S2) which contraindicate classic treatment using cholinesterase inhibitors [26, 27].

In two cases, the results of ES confirmed a clinical diagnosis when initial genetic testing using Sanger sequencing was reported as negative. The first case involved a 37-year-old male with ectodermal dysplasia, a cleft palate, right 3/4 toe syndactyly, nail dysplasia and tooth enamel dysplasia. No pathogenic variants had been identified on prior clinical testing for *TP63* variants by Sanger sequencing. Parent-child ES trio analysis identified a novel partial exon 11 deletion within the *TP63* gene (c.1350-75_1492del). Subsequent RNA analysis confirmed the absence of exon 11 from the *TP63* transcript. This variant has not been previously reported, but an intron 10 acceptor site variant has been previously observed in a similarly affected patient [28]. The second case was of a child with neonatal hypertrophic cardiomyopathy (deceased at 1 month of age). Prior to ES, clinical diagnostic testing by Sanger sequencing was undertaken for *PRKAG2* and was reported negative. Subsequent exome parent-child trio analysis identified a mosaic variant within the *PRKAG2* gene, c.1592G>A, p.(Arg531Gln), estimated to be present in 18% of reads (Additional file 2: Figure S2). The variant has been previously published as a cause of fatal congenital cardiac glycogenosis [29], and the severe HCM phenotype is consistent with this. The variant was considered a post-zygotic de novo mutation with low recurrence risk. Close re-inspection of Sanger traces showed the variant to be present (Additional file 2: Figure S2).

ES can also provide information on tissue-specific mosaicism. This is illustrated by a newborn female infant with an apparent dysmorphic syndrome, consisting of hypertelorism, epicanthic folds, cleft palate, 2/3 syndactyly, nail hypoplasia, abnormal eye movements and neurological problems. The child was dependent on a ventilator. Previous abnormalities had been detected during an anomaly scan at ~ 20 weeks gestation, and a sample taken by amniocentesis had been tested by aCGH and found to show no significant chromosomal imbalance. After the child was born, aCGH was discussed within the clinical team but was thought unnecessary due to the result at the time of amniocentesis. Parent-child trio ES analysis, utilising Exome Depth and skewed heterozygous allele frequencies in the child, identified a contiguous deletion and duplication on chromosome 17 (spanning multiple genes, approximately 6 Mb and 8 Mb respectively). A clinical audit confirmed the correct sample had been analysed and scored correctly on aCGH with the amniocentesis sample not identifying the imbalance. Fetal DNA (from blood) was then analysed by aCGH and this confirmed the result of the exome analysis: a mosaic deletion/duplication on chromosome 17.

In 5 cases (6.6% of those where ES completed), ES identified a known gene for a related phenotype to the presenting complaint. This is illustrated by a 2-year-old female patient with a diaphragmatic hernia, severe micrognathia, cleft palate, short thumbs, broad great toes, ventricular and atrial septal defects, and patent ductus arteriosus. The working clinical diagnosis of Fryns-like Syndrome was changed when a molecular diagnosis was made by ES, parent-child trio analysis revealing a de novo variant in *SF3B4* (c.1175dupC, p.(Pro393fs)), a gene in which pathogenic variants are known to cause Nager syndrome [30]. Other cases included a 12-year-old male with bilateral progressive hearing loss unresponsive to hearing aids and evidence of a distal axonal neuropathy for whom, within the spectrum of Charcot-Marie-Tooth disorders, a diagnosis of the rare X-linked recessive Cowchock syndrome was made after a c.1684A>G p.(Lys562Glu) variant in *AIFM1* [31] was identified in the proband and grandfather. In a further case, a 24-year-old woman with childhood-onset migraine, aura and possible migralepsy was found to have a variant in a known epilepsy gene, the chromatin modifier *CHD2* [32] (c.2402C>G p.(Thr801Arg)). An infant with suspected Ohtahara syndrome who presented with seizures, microcephaly and increased distal tone (deceased aged 1 month) was found to have a BRAT-1-related lethal neonatal rigidity and multifocal seizure syndrome [33] due to compound heterozygous variants (*BRAT1* c.294dupA p.(Leu99fs); heterozygous deletion of 3′ end of exon 14). Finally, a working diagnosis of Mowat Wilson syndrome was changed to Pitt-Hopkins syndrome in a 2.5-year-old male with severe developmental delay, no speech, microcephaly, poor weight gain and a happy demeanour after a de novo mutation was identified in *TCF4* [34] (c.1486G>C, p.(Gly496Arg)).

## Discussion

We have described our experience of establishing a multi-disciplinary team format for the application of NGS in a clinical setting, using a prospective ES case series to illustrate the operation and results of this approach. Our findings demonstrate the value of a multidisciplinary evaluation and consensus-based decision-making by a team within a clinical environment, complementing and informing related but distinct experience reported elsewhere such as the Genome Clinic Task Force (Geneva) [6], Genomic Consultation Service (British Columbia) [35], Individualised Medicine Clinic (Mayo) [36] and Undiagnosed Disease Network (NIH) [9]. Here we have shown how this approach can be implemented at a local level within a national health service, the impact of case selection, sequencing strategy and evaluation of results, and the health economics of such a format. The GM-MDT was set up in a translational research environment as an innovative approach not yet established in the UK at that time, enabled and supported by the Oxford NIHR BRC. It has now been successfully rolled out and embedded within the NHS with trio recruitment wherever possible based on the high diagnostic rate seen here. One area of expansion of MDT function is increased frequency to weekly meetings to enable discussion of variant classification in the context of clinical phenotype when considering results. Multi-disciplinary genetic service delivery is currently advocated in the UK [37] and has been enabled by the establishment of 13 regional NHS Genomic Medicine Centres (GMCs) with the implementation of an NHS Genomic Medicine Service in progress [38]. The Oxford GM-MDT has been instrumental in defining care pathways (for example involving incidental findings [11]) and guided on local policy for the 100,000 Genomes Project [38] where the GM-MDT has been adopted and implemented for NHS cases undergoing NGS at our Genomic Medicine Centre, highlighting the translational utility of this multi-disciplinary team format. Building in representation across a breadth of clinical specialties and researchers has meant that from the outset the approach taken was guided by representatives of the specialities the project sought to include, and has provided an ongoing forum for engagement and education.

The results presented here highlight how diagnosing the aetiology of a rare genetic disorder can be a challenge: the presenting condition may have a plethora of differential diagnoses; subtle or absent phenotypes may exclude a condition within the initial clinical assessment; and false negative molecular results may have been previously reported leading the referring clinician to consider other causes. Indeed, the limitations of exome sequencing should also be considered in this context. While the capture and sequencing performance of this assay are consistent with previous reports [39], regions of low or inconsistent coverage could result in reduced sensitivity. The observed diagnostic rate is comparable to previous reports [1, 2, 4, 8] but we recognise the potential for further diagnostic yield from unsolved clinical exomes through research analyses [40] and the current challenge of assigning pathogenicity [16, 41] such that a putative molecular diagnosis frequently has significant caveats. Identified variants for which a clinical report was issued ranged from recognised and reported pathogenic variants through to variants where there was sufficient evidence to warrant follow-up within individual families but not sufficient evidence to categorically state the identified variant was the sole cause of the condition.

Given the phenotypic heterogeneity commonly encountered in suspected rare genetic disease, an important aspect of ES and GS is the ability to simultaneously screen genes associated with the suspected condition as well as the differential diagnoses. A resulting change in clinical diagnosis may result, as illustrated by our findings with *SF3B4* and Nager syndrome. With NGS panel testing, ES and GS becoming part of routine clinical practice, the diagnostic odyssey associated with serial testing of candidate genes is becoming significantly reduced [42, 43]. ES and GS are not encumbered by the restricted size of targeted capture panels, which may result in variability between different targeted gene panels for heterogeneous conditions. In our dataset, we found that exome analysis provides further informativeness by enabling interrogation of large gene panels for SNVs and small copy number changes simultaneously, illustrated by a case where a molecular diagnosis of BRAT-1-related lethal neonatal rigidity and multifocal seizure syndrome was made on parent-child trio analysis involving a single nucleotide deletion (c.294dupA p.(Leu99fs)) and heterozygous deletion of the 3′ end of exon 14 within the *BRAT1* gene, with clinical impact for subsequent reproductive choices by the parents. One important caveat is that this approach requires selection of cases/inheritance patterns where the large yield of variants of uncertain significance arising from testing large gene panels can be offset, for example in trios where de novo, compound heterozygous or homozygous variants can be prioritised. Current limitations in terms of sensitivity and specificity are also recognised for the detection of CNVs using ES [44].

Two separate referrals from the GM-MDT showed exome sequencing to be more sensitive than traditional Sanger sequencing for certain types of variant, identifying pathogenic variants in *TP63* and *PRKAG2* involving a partial exon deletion and a mosaic missense variant respectively. Our finding with *TP63* illustrates how ES can increase the sensitivity of existing diagnostic molecular tests and highlight new variant types in known disease-causing genes. The case involving *PRKAG2* highlights not only the informativeness of NGS but also the importance of read depth to provide sufficient sensitivity for such de novo/mosaic mutations. This result has changed local laboratory practice such that severe early-onset cases of HCM are now analysed by visual inspection of Sanger traces rather than by automated calling when testing *PRKAG2*, but with NGS as the preferred method.

Likely tissue-specific mosaicism involving a large structural variant was also revealed by ES in an infant with dysmorphism and congenital abnormalities following a normal prenatal aCGH result from amniocentesis, postnatal ES detecting the cytogenetic abnormality on chromosome 17. This has changed local clinical practice such that aCGH can now be requested after the child has been born to exclude tissue mosaicism that may give rise to a false negative prenatal amniocentesis test.

Discussions between clinicians and scientists at the GM-MDT also facilitated the detection of pathogenic variants within recently identified disease-causing genes. Indeed, a pathogenic variant involving a *CTPS1* splice site [21] was published while the DNA samples were being sequenced and later proved to be the cause of the child’s primary immune deficiency and help direct patient management [22]. This example illustrates how the integration of multiple disciplines can help target analysis on new disease-associated genes and how responsive exome and genome NGS analysis can be. Ensuring disease-specific knowledge of genetic aetiology is up to date is essential to maximise the informativeness of exome and genome-scale data and current large-scale initiatives such as the 100,000 Genomes Project and the PanelApp (https://panelapp.genomicsengland.co.uk/) can play a critical role in enabling the creation and maintenance of such a relevant and current knowledge base of disease-causing genes. Given such knowledge is being acquired over time, the burden on analysts to reanalyse variants within these newly discovered genes is likely to be substantial if there is insufficient informatics support, highlighting the importance of promoting efforts to establish an automated approach that may be facilitated by a cross-disciplinary MDT.

While we observed a higher molecular diagnostic rate where selection of cases was amenable to a trio design, in a number of instances ES of a singleton case was successful. The latter included instances where parents were consanguineous and little additional power was felt to accrue from sequencing the parents with significant cost saving. However, in this case, the analysis assumes a recessive condition within a region of homozygosity and not a de novo variant. Had the targeted analysis not found a pathogenic variant, subsequent analysis could have been hindered by the absence of ES data from the parents. Overall, adopting a trio-based strategy where possible is felt to have significant advantages, notably for identification of de novo dominant and compound heterozygous pathogenic variants [45]. Indeed, in adult-onset autosomal dominant conditions such as cardiomyopathy, ES/GS approaches in familial disease have given much lower yields than in the selected trios studied here [45].

In terms of the costs associated with the GM-MDT, if for example one case involves 3 exomes being sequenced as a trio, this would cost approximately £2160 per case which includes MDT costs, exome sequencing and analysis. In the future, our cost analysis could be helpful in the context of GS, as the GM-MDT costs could be combined with GS rather than ES costs.

## Conclusions

In this paper, we have described our experience with a newly established GM-MDT in the setting of a consecutive ES case series that adds to the body of evidence supporting a multi-disciplinary format for both selection of cases and evaluation of results when applying NGS in the clinic as well as specific implications for practice arising from a diverse case series. While GS is anticipated to replace ES as the standard NGS-based test for rare genetic disease, the lessons learnt from the application of ES continue to inform decision-making in both case selection as well as subsequent analysis and interpretation [46, 47].

## Additional files

Additional file 1: **Table S1.** Membership of GM-MDT. **Table S2.** Description of cases where exome sequencing completed including demographics, phenotyping and results. **Table S3.** Estimation of costs for GM-MDT (A) Time and costs for the MDT over the 10 month period; (B) Sensitivity analysis on MDT costs. **Table S4.** Exome sequencing and analysis costs. (XLSX 62 kb)

Additional file 2: **Figure S1.** Clinical phenotypes for all cases referred to GM-MDT. Figure S2. Mosaic PRKAG2 variant c.1592G>A, p.(Arg531Gln). (PDF 514 kb)

## Data Availability

Variants can be freely accessed at ClinVar (https://www.ncbi.nlm.nih.gov/clinvar/) accession numbers SCV000926196-SCV000926215 inclusive. For 18 cases where diagnostic results were reported clinically and appropriate consent was in place, VCF files and corresponding metadata have been deposited in the European Genome-phenome Archive (EGA) under accession number EGAS00001003771.

## Abbreviations

aCGH: Array comparative genomic hybridization
ACMG: American College of Medical Genetics and Genomics
BRC: Oxford Biomedical Research Centre
DDD: Deciphering Developmental Disorders Project
ES: Exome sequencing
ExAC: Exome Aggregation Consortium
GM-MDT: Genomic multi-disciplinary team
GS: Genome sequencing
MGAC: Molecular Genetic Analysis and Clinical studies of Individuals and Families at Risk of Genetic Disease
NGS: Next-generation sequencing
NIHR: National Institute of Health Research
OUH: Oxford University Hospitals
WHG: Wellcome Centre for Human Genetics
