# Supplementary material for: Implementation of a genomic medicine multi-disciplinary team approach for rare disease in the clinical setting: a prospective exome sequencing case series
Source: Genome Med. 2019 Jul 25;11:46. doi: 10.1186/s13073-019-0651-9 (PMC6659244; doi:10.1186/s13073-019-0651-9)
Supplement: Supplementary file 2 — Figure S1. Clinical phenotypes for all cases referred to GM-MDT. Figure S2. Mosaic PRKAG2 variant c.1592G>A, p.(Arg531Gln). (PDF 514 kb) [file 13073_2019_651_MOESM2_ESM.pdf]

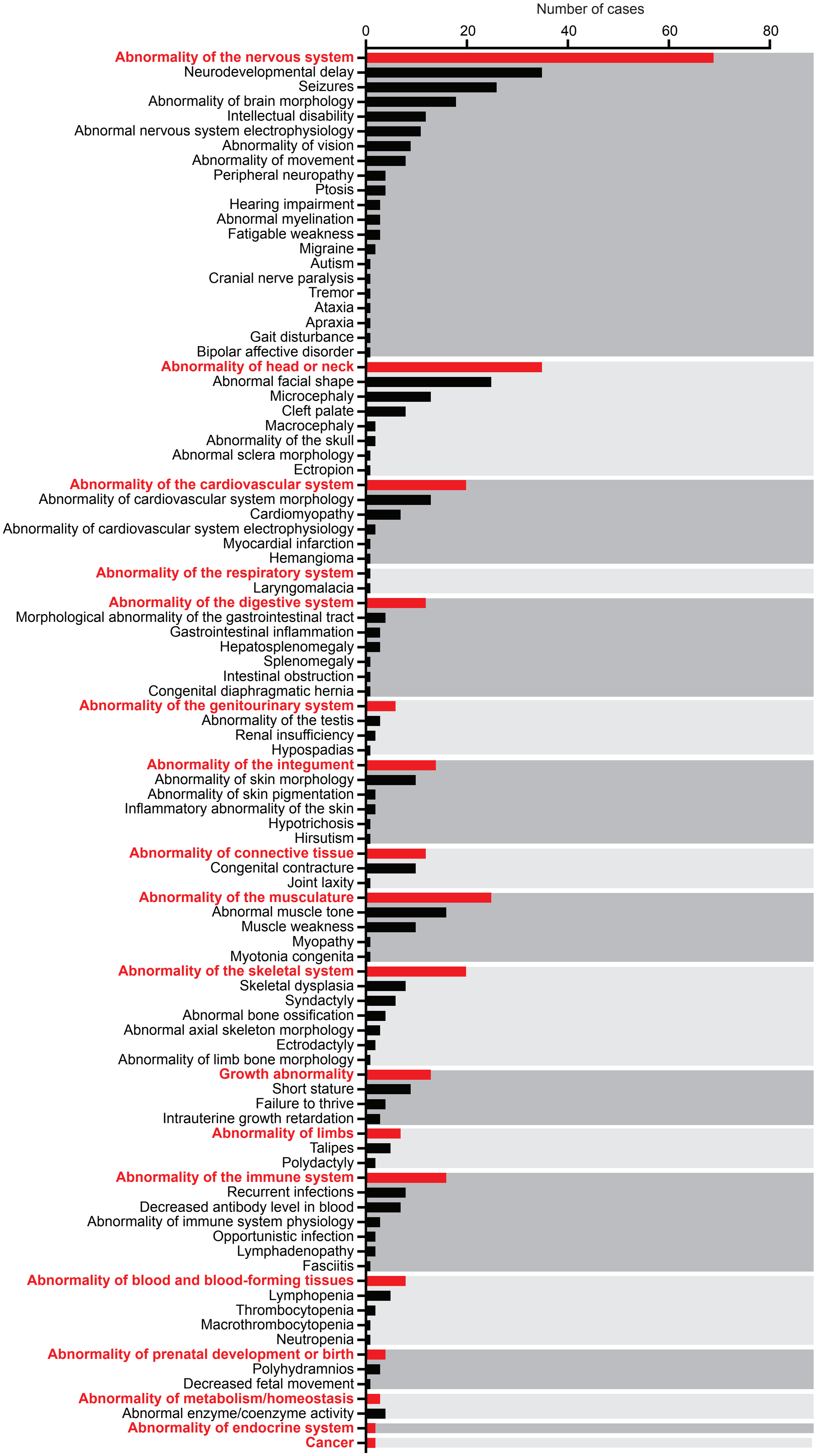

Supplementary Figure 1. Clinical phenotypes for all cases referred to GM-MDT (n=132). Human phenotype ontology terms used.

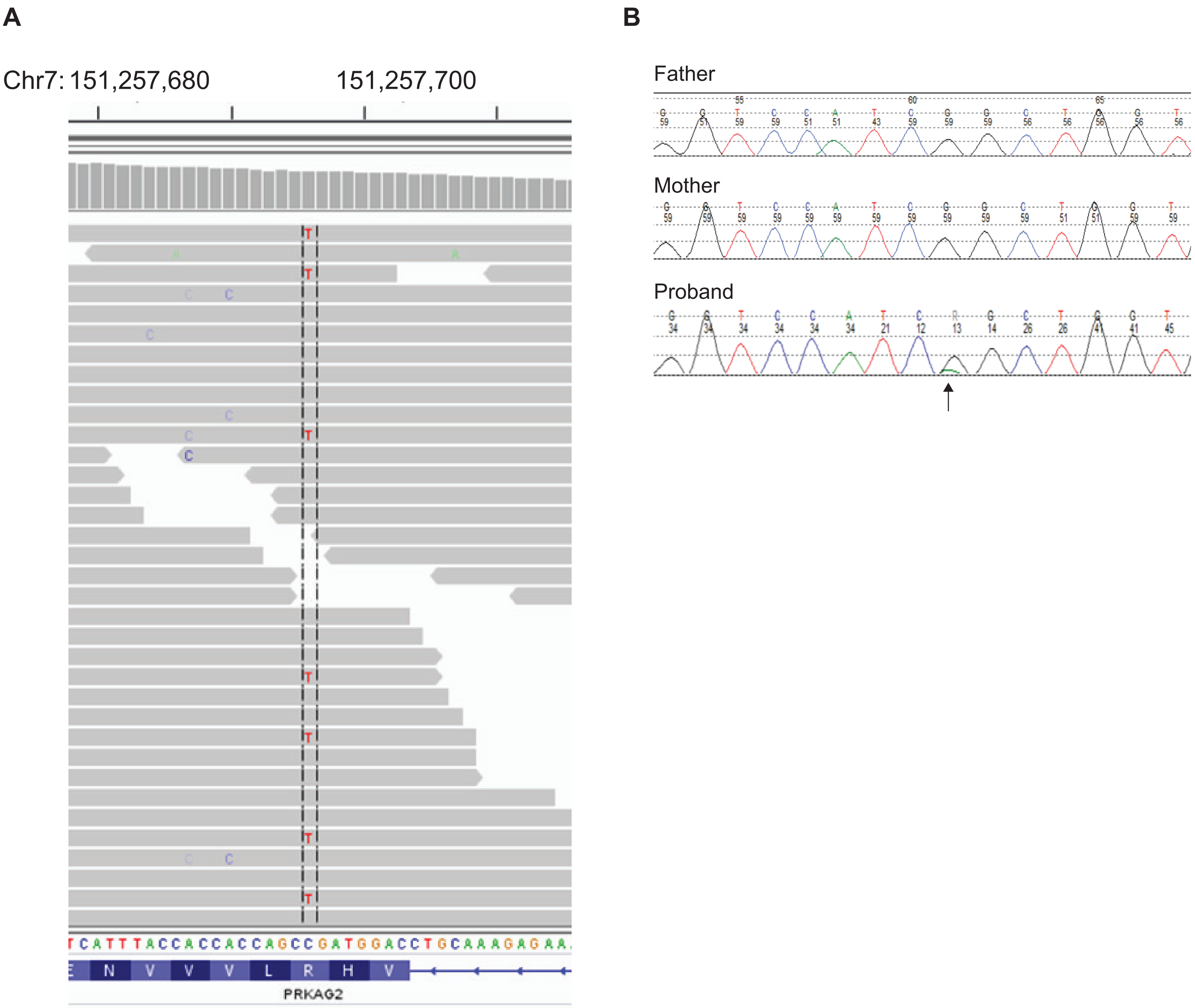

Supplementary Figure 2. Mosaic *PRKAG2* variant c.1592G>A, p.(Arg531Gln). (**A**) Aligned ES reads for the proband visualised in IGV show the variant base change was present in 22/122 (18%) of Illumina reads mapping to the *PRKAG2* gene (5’ to 3’ runs right to left). (**B**) Sanger sequencing confirmation (the reverse complement of the sequence in panel A, 5’ to 3’ from left to right) illustrates the presence of the variant adenosine base (indicated by the arrow).
